# Analysis of enhanced CT imaging signs and clinicopathological prognostic factors in hepatoid adenocarcinoma of stomach patients with radical surgery: a retrospective study

**DOI:** 10.1186/s12880-023-01125-z

**Published:** 2023-10-26

**Authors:** Xin-Yue Yan, Hai-Yue Ju, Fang-Jing Hou, Xiao-ting Li, Ding Yang, Lei Tang, Ya-Kun Wang, Zhong-Wu Li, Ying-Shi Sun, Shun-Yu Gao

**Affiliations:** 1 Department of Radiology, Key Laboratory of Carcinogenesis and Translational Research (Ministry of Education/Beijing), Peking University Cancer Hospital & Institute, No. 52 Fu Cheng Road, Beijing, Hai Dian District 100142 China; 2 Department of Digestive Oncology, Key Laboratory of Carcinogenesis and Translational Research (Ministry of Education/Beijing), Peking University Cancer Hospital & Institute, No. 52 Fu Cheng Road, Beijing, Hai Dian District 100142 China; 3 Department of Pathology, Key Laboratory of Carcinogenesis and Translational Research (Ministry of Education/Beijing), Peking University Cancer Hospital & Institute, No. 52 Fu Cheng Road, Beijing, Hai Dian District 100142 China

**Keywords:** Hepatoid adenocarcinoma, Gastric cancer, Prognosis factor, Computed tomography

## Abstract

**Background:**

To investigate the association between CT signs and clinicopathological features and disease recurrence in patients with hepatoid adenocarcinoma of stomach (HAS).

**Methods:**

Forty nine HAS patients undergoing radical surgery were retrospectively collected. Association between CT and clinicopathological features and disease recurrence was analyzed. Multivariate logistic model was constructed and evaluated for predicting recurrence by using receiver operating characteristic (ROC) curve. Survival curves between model-defined risk groups was compared using Kaplan–Meier method.

**Results:**

24(49.0%) patients developed disease recurrence. Multivariate logistic analysis results showed elevated serum CEA level, peritumoral fatty space invasion and positive pathological vascular tumor thrombus were independent factors for disease recurrence. Odds ratios were 10.87 (95%CI, 1.14–103.66), 6.83 (95%CI, 1.08–43.08) and 42.67 (95%CI, 3.66–496.85), respectively. The constructed model showed an area under ROC of 0.912 (95%CI,0.825–0.999). The model-defined high-risk group showed poorer overall survival and recurrence-free survival than the low-risk group (both *P* < 0.001).

**Conclusions:**

Preoperative CT appearance of peritumoral fatty space invasion, elevated serum CEA level, and pathological vascular tumor thrombus indicated poor prognosis of HAS patients.

**Supplementary Information:**

The online version contains supplementary material available at 10.1186/s12880-023-01125-z.

## Background

Hepatoid adenocarcinoma is a primary tumor with abnormal differentiation of hepatocytes in extrahepatic organs. The incidence of hepatoid adenocarcinoma is relatively rare. It mainly occurs in the gastrointestinal tract, especially in the stomach [1]. Hepatoid adenocarcinoma of stomach (HAS) is often accompanied by increased serum α-fetoprotein (AFP) but with nonspecific clinical symptoms. Currently, the underlying mechanisms regarding the development and recurrence of HAS are unknown. HAS shows both adenocarcinoma and hepatocyte-like differentiation. It has been hypothesized that the stomach and liver originate from the endoderm and the primitive foregut during embryonic development. Therefore, these two organs are closely related in histology and embryology [2–4]. Some gastric tumor cells can differentiate into hepatocytes in two histological manifestations: one is the medullary portion, which is characterized by hepatocellular carcinoid differentiation of gastric cancer cells, arranged in a manner similar to primary liver cancer; the other is the well differentiated papillary or tubular area, which shows tubulopapillary architecture and is remarkable for marked subnuclear vacuolization or clear cytoplasm resembling those in the primitive gut. Since the adenocarcinoma usually presents as gastric adenocarcinoma with enteroblastic differentiation and atypical biological behavior, the diagnosis is not sufficient by morphology alone. The diagnosis of this neoplasm is required resorting to immunohistochemistry [5, 6].

HAS is highly malignant, invasive, and prone to lymph node and liver metastasis [7]. At present, surgery is the main clinical treatment for HAS [8]. After treatment, the prognosis is various in different HAS patients. It is even with a worse prognosis than common gastric adenocarcinoma. Previous imaging analysis of HAS was mainly compared with that of ordinary gastric cancer, and the results showed that that size of lesion, serum AFP level, M stage, and degree of tumor enhancement on CT are independent factors for differentiating HAS from gastric adenocarcinoma [9–11]. HAS is often characterized by eccentricity and uneven thickness of the gastric wall with a strong tendency to liver and lymph node metastasis. However, it is a lack of the study on the image signs combined with clinical and pathological characteristics with the prognosis of HAS patients. In this study, the patients with HAS who underwent radical resection were taken as the research objects. The study proposed to comprehensively analyze multi-phase enhanced CT imaging signs before treatment combined with the clinical features and postoperative pathological results according to the prognosis of the patients after radical resection, so as to define the key prognostic factors for HAS.

## Methods

### Patients

**Fig. 1:**
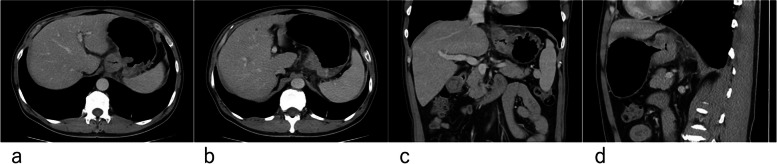
**a**-**d**, a patient with poorly differentiated HAS at the junction of esophagus and stomach. Enhanced CT showed full-thickness involvement of the gastric wall with the involvement of the surrounding fat space, increased density of the surrounding fat space, and visible cord shadow. The postoperative pathology showed vascular tumor thrombus was positive. Multiple liver metastasis occurred 18 months after radical surgery

**Fig. 2:**
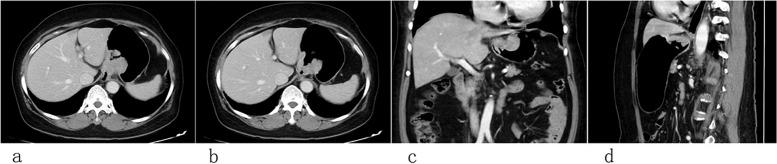
**a**-**d**, a patient with poorly differentiated HAS at the junction of esophagus and stomach. Enhanced CT showed full-thickness involvement of the gastric wall without the involvement of the surrounding fat space, clear surrounding fat space. The postoperative pathology showed vascular tumor thrombus was positive. No obvious recurrence and metastasis were found in the follow-up period after radical surgery, and the tumor free survival period was 38.4 months

This study was approved by our institutional review board, and the patient informed consent was waived due to the retrospective design. Complete clinical information, pre-treatment enhanced CT images and postoperative pathological data of patients with HAS who were admitted to Peking University Cancer Hospital from January 2015 to December 2018 after radical resection were retrospectively collected. The eligibility criteria in the study included: (1) pathologically and immunohistochemically confirmed HAS: typical HAS consists of large polygonal eosinophilic hepatocellular carcinoma-like cells; gastric adenocarcinoma with enteroblastic differentiation showed obvious sub nuclear vacuolation or clear cytoplasm similar to the primitive gut, and at least one of the immunohistochemical markers AFP, GPC3 and SALL4 was applied; (2) underwent radical surgery directly or after neoadjuvant chemotherapy; (3) underwent multi-phase enhanced abdominal CT examination with hypotonic drug injection and gastric gas filling before treatment. The imaging data were complete, and lesions larger than 5 mm were obtainable, measurable and reconstructable on CT images. For patients who received neo-adjuvant chemotherapy, all analyses were based on the CT images acquired before systemic treatment (Figs. 1, 2); (4) had complete clinical data and postoperative pathological results; (5) had regular and complete follow-up results after surgery, and the database was updated on February 28, 2022. During follow-up, patients had serum tumor markers test (including CEA and CA19-9), gastric endoscopy, upper gastrointestinal contrast examination, chest and abdominal CT examination and were seen every 3 months for 2 year, every 6 months for an additional 3 years, and then annually thereafter. Patients with the following conditions were excluded from our study: (1) the enhanced CT imaging data before treatment is incomplete; (2) the images are insufficient for further analysis due to small lesions, poor image quality or dissatisfied gastric filling; (3) the patients had other malignant tumors; (4) the patients failed to undergo radical surgical resection; (5) irregular postoperative follow-up information or not reliable follow-up results.

### CT Technique

All patients received hypotonic gastric gas filling plain scanning and multi-phase enhanced CT scanning before surgery. The CT scans were obtained using GE Light Speed VCT or Philips Brilliance 64-slice spiral CT scanners. After fasting for more than 6 h, patients were intramuscularly injected raceanisodamine hydrochloride injection 10 min before the examination. After achieved hypotonic drug effect, that is when patients felt dry mouth, 2 packets of gas-producing powder were orally administered to obtain gastric distention immediately before the plain scanning examination.

After plain CT scan of the abdomen, 90–100 ml (1.5mgI/kg) of non-ionic contrast agent (iohexol 300mgI/ml) was intravenously injected at one time at the 2.5–3.5 ml/s injection flow rate. When the descending aorta reaches 100 HU after contrast agent injection, the arterial phase, portal venous phase and delayed phase enhanced scan were performed with delays of 10 s, 90 s and 180 s by using automatic trigger scanning technology. The images were reconstructed according to the standard algorithm for conventional images and thin-slice images with a slice thickness of 5.0 mm and 0.625 mm, with a window width of 350 HU and a window level of 45 HU. Coronal and sagittal multiplane image reconstructions were routinely performed. All images were transmitted and stored in DICOM format.

### Clinical data collection

We collected pre-treatment clinical information including: gender, age, height, weight, body mass index (BMI), main symptoms, serum α-fetoprotein (AFP), carcinoembryonic antigen (CEA), carbohydrate antigen 19–9 (CA199), carbohydrate antigen 125 (CA125) and carbohydrate antigen 724 (CA724) level, treatment methods, neoadjuvant therapy before and after surgery. Postoperative pathological data include: differentiation degree, maximum diameter, Lauren classification, pathological stage, T stage, N stage, vascular tumor thrombus, nerve invasion. And immunohistochemical results include: AFP, BRCA-1, CD44, Cmet, EGFR, ERCC- 1, Hep, HER2, Ki-67, P53, SALL4, MLH1/2/6, PD-L1.

### Image evaluation

Two experienced radiologists with 6- and 15- years’ experience in abdominal imaging diagnosis reviewed CT images independently through the workstation using a unified soft tissue window (window width 350 HU, window level 45 HU), and adjusted the window width and level appropriately to best display the details of the structures to be observed. When measuring the CT value, a circular region of interest (ROI) and manual delineation were used to cover the tumor tissue as much as possible, avoiding blood vessels, necrotic tissue and artifacts. The CT values of gastric hepatoid adenocarcinoma lesions in different phases, abdominal aorta and largest lymph node at the same level were measured by copying and pasting through ROI of the same size. Metastatic lymph nodes were defined as peripheral lymph nodes that were more than 8 mm in diameter. The relative enhancement degree of tumor and metastatic lymph node in arterial phase, venous phase and delayed phase, tumor enhancement type, and the ratio of CT value of tumor to abdominal aorta (T/A) were calculated. Lymph node measurements were not performed in patients without metastasis.

**Fig. 3:**
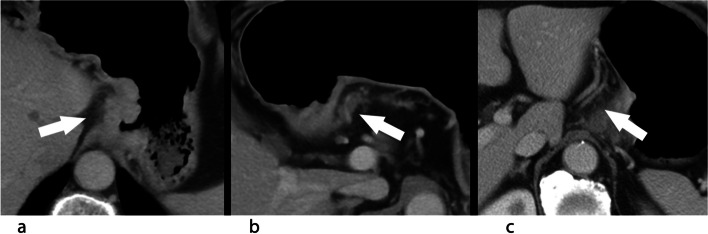
CT features of infiltrating peritumoral adipose space. **a** substantial density increase; **b** nodular intensities; **c** irregular linear opacities

The enhanced CT images of the portal venous phase were selected to evaluate the following signs of gastric hepatoid adenocarcinoma lesions and the largest metastatic lymph nodes: location, length, thickness, Borrmann classification, serosal surface invasion (disappearance and blurring of normal structures on the serosal surface), surrounding fat space (infiltrating/non-infiltrating; the CT findings of infiltrating peritumoral adipose space include substantial density increase of the peritumoral fat space or the presence of nodular or irregular linear opacities in the peritumoral fatty space (Fig. 3]), T stage, vascular invasion (extramural blood vessels around the tumor, vascular contour/enhancement changes), vascular tumor thrombus (the presence of tumor thrombus with low density and abnormal enhancement filling defect in local vein widening), necrosis, distant metastasis, stage of metastatic lymph node, long diameter, the short diameter of the largest metastatic lymph node, extracapsular invasion of lymph nodes and lymph node necrosis.

### Patient follow-up

The patients were routinely rechecked and assessed for serum tumor markers, plain chest CT scan and enhanced abdominal CT scan every 3 to 6 months after radical surgery. Our follow-up adopts a combination of inpatient review, outpatient review, telephone and letter, and the deadline for follow-up is December 30, 2021. During follow-up, anastomotic tumor recurrence, regional or distant lymph node metastasis, other organ metastasis and death were defined as recurrence. Anastomotic recurrence required pathological confirmation by endoscopy or needle biopsy. The lymph nodes and other organ metastasis were diagnosed by needle biopsy or typical imaging. The recurrence-free survival was calculated from the time of enhanced CT examination before treatment to the time of disease progression or death, or censored at the last follow-up. The overall survival was calculated from the time of enhanced CT examination before treatment to the time of death, or censored at the last follow-up.

### Statistical analysis

Continuous variables were described as means ± standard deviation; categorical variables were defined as numbers and proportions. Variables between groups were compared using independent t-test/Mann–Whitney test or Chi-square test/Fisher‘s exact test. Multivariate logistic regression with a step-wise method was performed to screen for independent factors associated with disease recurrence, and a predictive model was constructed accordingly. The receiver operating characteristic curve was performed. And the cutoff was determined using the maximum of the Youden index. Patients were divided into high or low risk group according to the cutoff. Kaplan–Meier method with log-rank estimates were used to compare the survival curves for recurrence-free survival and overall survival. Inter-rater agreement was assessed using intra-class correlation coefficient, 0.00–0.20, 0.21–0.40, 0.41–0.60, 0.61–0.80 and 0.81–1.00 were considered as poor, fair, moderate, substantial and perfect agreement, respectively.

All statistical analysis was conducted using SPSS (version 22.0) and R package (version 4.1.2). A two-sided *P* less 0.05 was considered statistically significant.

## Results

### Patients

During the period from January 2015 to December 2018, 69 patients were initially diagnosed and treated for HAS in our hospital, including 13 patients without radical surgical resection, 1 patient with esophageal cancer, 1 patient died due to serious postoperative complications, 1 patient without preoperative enhanced CT imaging data, 2 patients with lesions that could not be accurately measured due to enhanced CT image artifacts and 2 patients with hardly measured small lesion. Thus, a total of 20 patients were excluded. Finally, a total of 49 patients with HAS undergoing radical surgical resection were included in this analysis (43 males and 6 females, aged from 44 to 76 years, with an average of 60.49 ± 7.64 years). Preoperative Borrmann's classification included 1 patient of type I, 0 patient of type II, 47 patients of type III, and 1 patient of type IV (Table 1).

**Table 1:** Characteristics of 49 hepatoid adenocarcinoma of the stomach patients

Characteristic	No. of patients (%)
Sex	
Male	43(87.8%)
Female	6(12.2%)
BMI	
< 18.5	4(8.2%)
18.5–24	20(40.8%)
> 24	25(51.0%)
Primary site of the tumor	
Gastroesophageal junction	17(34.7%)
Body of stomach	9(18.4%)
Antrum of stomach	23(46.9%)
Borrmann type	
I	1(2.0%)
III	47(96.0%)
IV	1(2.0%)
The serum level of AFP	
≤ 450 ng/ml	39(79.6%)
> 450 ng/ml	10(20.4%)
The serum level of CEA	
≤ 5.0 ng/ml	26(53.1%)
> 5.0 ng/ml	23(46.9%)
The serum level of CA199	
≤ 51.51 ng/ml	44(89.8%)
> 51.51 ng/ml	5(10.2%)
The serum level of CA724	
≤ 5.0 ng/ml	36(73.5%)
> 5.0 ng/ml	13(26.5%)
The serum level of CA125	
≤ 50.0 ng/ml	48(98.0%)
> 50.0 ng/ml	1(2.0%)
Histopathology	
Well differentiated adenocarcinoma	10(20.4%)
Moderately differentiated adenocarcinoma	5(10.2%)
Poorly differentiated adenocarcinoma	34(69.4%)
Lauren's classification	
Intestinal type	31(63.3%)
Diffuse type	3(6.1%)
Mixed type	15(30.6%)
T classication^a^	
T1	3(6.1%)
T2	9(18.4%)
T3	27(55.1%)
T4	10(20.4%)
N classication^a^	
N0	3(6.1%)
N1	5(10.2%)
N2	26(53.1%)
N3	15(30.6%)
Clinical stage^a^	
IA	2(4.0%)
IB	1(2.0%)
IIA	8(16.0%)
IIB	9(2.0%)
IIIA	17(18.4%)
IIIB	7(14.3%)
IIIC	2(4.1%)
IV	3(6.1%)
Pathological vascular tumor thrombus	30(61.2%)
CT finding of vascular tumor thrombus	0(NA)
Neoadjuvant chemotherapy regimens	
Yes	19(38.8%)
No	30(61.2%)

^a^Assessed according to the 8th AJCC staging system

### Characteristics associated with disease recurrence

24(49.0%) patients developed disease recurrence, 12(24.7%) patients of them suffered hepatic metastasis, 5(10.2%) patients developing peritoneal and/or retroperitoneal lymph node metastasis, 1(2.0%) patient developing peritoneal metastasis, 1(2.0%) patient developing lung metastasis and 5(10.2%) patients of them suffered death. The median of follow-up period was 45 months (95% CI, 39–52).

Substantial to perfect inter-rater agreement were obtained for CT features (ICC from 0.785 to 0.969). Univariate analysis results showed that disease recurrences were significantly associated with elevated serum CEA level, cardia, positive serosal surface, peritumoral fatty space invasion, higher T stage, larger long and short lymph node diameter, involvement of outside the lymph node capsule, positive pathological vascular tumor thrombus and nerve invasion (Table 2).

**Table 2:** Analysis of characteristics associated with disease recurrence

		**Disease recurrence**		
		**No (*n* = 24)**	**Yes (*n* = 25)**	***P***
Vascular tumor thrombus	-	16(66.6)	3(12.0)	< 0.001
	+	8(33.3)	22(88.0)	
Nerve invasion	-	18(75.0)	7(28.0)	< 0.001
	+	6(25.0)	18(72.0)	
CEA	≤ 5.0 g/ml	18(75.0)	8(32.0)	0.015
	> 5.0 g/ml	6(25.0)	17(68.0)	
Serosal surface	-	19(79.2)	10(40.0)	0.005
	+	5(20.8)	15(60.0)	
Peritumoral fatty space	-	17(70.8)	8(32.0)	0.007
	+	7(29.2)	17(68.0)	
cT stage	2	4(16.7)	1(4.0)	0.031
	3	15(62.5)	10(40.0)	
	4	5(20.8)	14(56.0)	
Location	Esophagogastric junction/cardia	5(20.8)	12(48.0)	0.046
	Gastric body(Lesser curvature and antrum)	19(79.2)	13(52.0)	
Long diameter of lymph node		16.31 ± 6.36	24.84 ± 11.22	0.011
Short diameter of lymph node		11.69 ± 5.08	19.16 ± 8.17	0.009
Extracapsular invasion of lymph node	-	24(100.0)	16(64.0)	0.002
	+	0(0.0)	9(36.0)	

Multivariate logistic analysis results showed that elevated serum CEA level, peritumoral fat space invasion and positive pathological vascular tumor thrombus were significantly independent factors for disease recurrence of HAS patients after radical surgical resection, with OR were 10.87 (95%CI, 1.14–103.66, *P* = 0.003), 6.83 (95%CI, 1.08–43.08, *P* = 0.041) and 42.67 (95%CI, 3.66–496.85,* P* = 0.038), respectively (Table 3). Among the 24 patients with infiltrating peritumoral fat space, during the follow-up period, 17 patients had disease recurrence, including anastomotic tumor recurrence in 0 patients, regional or distant lymph node metastasis in 3 patients, other organ metastasis in 12 patients (liver:6, lung:1, rectum and rectovesical pouch:1, liver and pancreas:1, liver and bile duct:1, liver and lung:1), and 10 patients unfortunately succumbed to cancer. In addition, no statistical significance was observed in other CT characteristics, such as the CT value, relative enhancement degree of tumor and metastatic lymph node in arterial phase, venous phase and delayed phase, tumor enhancement type, and the ratio of CT value of tumor to abdominal aorta (T/A) (Table 4).

**Table 3:** Multivariate analysis results according to disease recurrence

	**β**	**OR (95%CI)**	***P***
Vascular tumor thrombus ( +)	3.75	42.67(3.66–496.85)	0.003
CEA(> 5.0 g/ml)	2.39	10.87(1.14–103.66)	0.038
Peritumoral fatty space ( +)	1.92	6.83(1.08–43.08)	0.041

**Table 4:** Analysis of CT characteristics associated with recurrence

		**Recurrence**		
		**No (*n* = 24)**	**Yes (*n* = 25)**	***P***
cN classication	0	9(37.5)	6(24.0)	0.305
	1	15(62.5)	19(76.0)	
Vascular invasion	0	22(91.7)	18(72.0)	0.138
	1	2(8.3)	7(28.0)	
Vascular tumor thrombus	0	24(100.0)	25(100.0)	NA
	1	0	0	
Necrosis	0	23(95.8)	25(100.0)	0.49
	1	1(4.2)	0(0.0)	
Short diameter		13.85 ± 3.96	16.26 ± 6.57	0.129
Long diameter		47.17 ± 17.01	54.86 ± 15.96	0.110
Lymph node necrosis	0	22(91.7)	17(68.0)	0.074
	1	2(8.3)	8(32.0)	
CT value in plain scan		35.52 ± 10.45	38.26 ± 9.05	0.333
ROI CT value in plain scan		30.23 ± 9.47	35.62 ± 8.75	0.044
Aorta CT value in plain scan		43.17 ± 7.34	40.58 ± 9.43	0.291
Lymph node CT value in plain scan		28.35 ± 14.27	35.71 ± 13.10	0.116
CT value in arterial phase		78.85 ± 21.07	80.78 ± 14.59	0.711
ROI CT value in arterial phase		72.63 ± 18.12	77.24 ± 13.11	0.311
Aorta CT value in arterial phase		139.38 ± 20.72	145.58 ± 21.92	0.314
Lymph node CT value in arterial phase		69.09 ± 18.38	74.18 ± 28.66	0.535
CT value in venous phase		78.31 ± 24.66	75.42 ± 19.36	0.649
ROI CT value in venous phase		72.15 ± 22.61	71.98 ± 19.87	0.978
Aorta CT value in venous phase		248.44 ± 50.37	265.42 ± 56.49	0.273
Lymph node CT value in venous phase		59.63 ± 20.41	73.50 ± 25.24	0.087
CT value in delayed phrase		68.71 ± 23.32	73.60 ± 20.08	0.435
ROI CT value in delayed phase		66.23 ± 22.63	71.22 ± 17.61	0.392
Aorta CT value in delayed phase		111.02 ± 33.03	110.08 ± 25.16	0.911
Lymph node CT value in delayed phase		43.20 ± 33.26	60.61 ± 23.81	0.065
Enhancement type	1	4(16.7)	3(12.0)	0.926
	2	12(50.0)	14(56.0)	
	3	8(33.3)	8(32.0)	
Relative CT value in arterial phase		0.15 ± 0.13	0.21 ± 0.18	0.172
Relative CT value in venous phase		0.32 ± 0.27	0.40 ± 0.27	0.299
Relative CT value in venous phase		0.36 ± 0.32	0.44 ± 0.30	0.347
Lymph node relative CT value in arterial phase		0.24 ± 0.06	0.27 ± 0.16	0.518
Lymph node relative CT value in venous phase		0.51 ± 0.12	0.47 ± 0.22	0.587
Lymph node relative CT value in delayed phase		0.59 ± 0.15	0.53 ± 0.25	0.403
Lymph node enhancement type	1	3	6	0.348
	2	7	4	
	3	5	9	

Substantial to perfect inter-rater agreement were obtained for CT characteristics (ICC were 0.735 to 0.995)

### Predictive model for disease recurrence

**Fig. 4:**
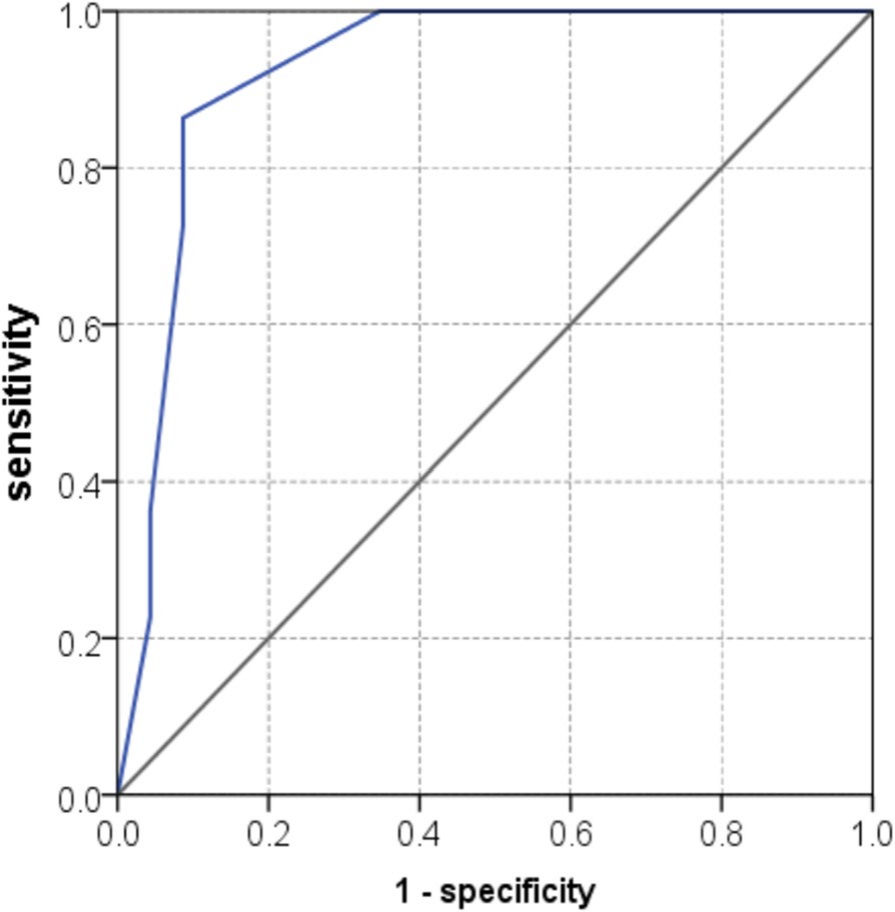
Receiver operating characteristic curve of the multivariate model for predicting disease recurrence

The multivariate model constructed accordingly showed an AUC of 0.912 (95%CI, 0.825–0.999) for predicting disease recurrence (Fig. 4). 25 patients with model predicted probability for disease recurrence larger than 40% (cut-off selected by ROC curve) were assigned in high -risk group, while 24 patients with probability ≤ 40% were assigned in low-risk group. The sensitivity, specificity and overall accuracy of the model for predicting disease recurrence were 84% (21/25), 83.3% (20/24) and 83.7% (41/49).

**Fig. 5:**
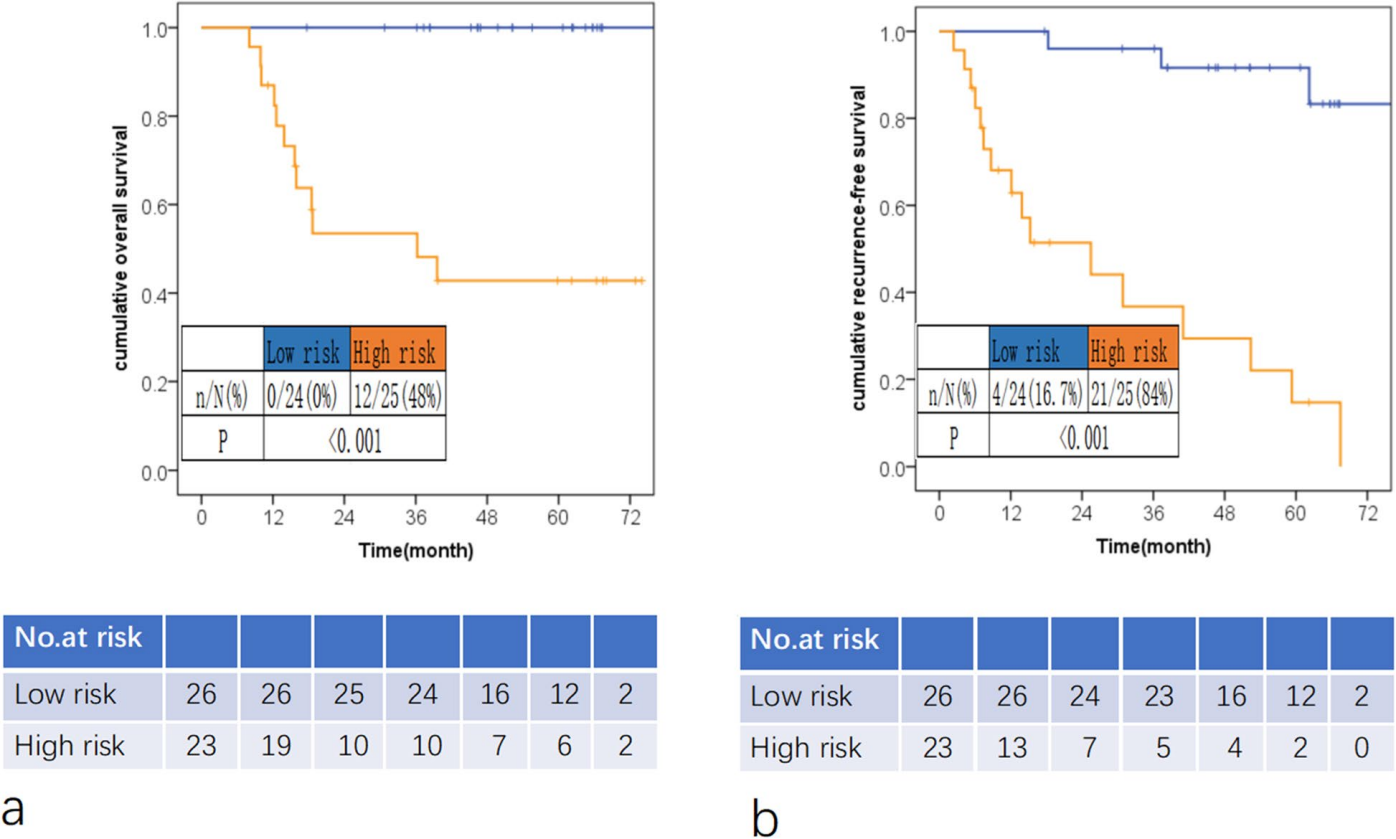
Kaplan–Meier curves of model defined risk groups according to overall survival **a** and disease recurrence survival **b**

Model-defined high-risk group showed significantly poorer overall survival and recurrence-free survival than the low-risk group, both *P* < 0.001 (Fig. 5).

### Effect of neoadjuvant chemotherapy on disease recurrence

Among the 30 patients without neoadjuvant chemotherapy, 12(40%) experienced disease recurrence; while among the 19 patients with neoadjuvant chemotherapy, 13(68.4%) experienced disease recurrence. There was no significant difference in the recurrence rate between patients who underwent neoadjuvant chemotherapy and those who did not. (*P* = 0.052).

In the 7 patients without adjuvant chemotherapy, 3(47.6%) experienced disease recurrence; while in the 42 patients with adjuvant chemotherapy, 22(52.4%) suffered disease recurrence. Adjuvant chemotherapy was not significant for disease recurrence, OR was 0.75(95%CI, 0.15–3.87), *P* = 0.73.

## Discussion

HAS have a high degree of malignancy. In our study, 34 patients (69.4%) were poorly differentiated cancers, and 10 patients (20.4%) were moderately differentiated cancers, most of which were poorly differentiated cancers, consistent with previous studies. The prognosis of HAS patient is poor, there is no complete personalized treatment plan for this subtype, the treatment plan of all cases in this study before and after surgery is similar to that of other common gastric cancers. Neoadjuvant treatment, which has no standard for HAS were recommended, either alone or combined, the prognosis of advanced HAS still remains obscure [8]. Further research is expected to obtain an accurate treatment plan for HAS, so as to improve the prognosis of HAS patient [12]. In this study, some pre-treatment CT imaging signs and clinical pathological factors were founded which would be used to predict the prognosis of the HAS patients.

Hepatocyte differentiation in gastric cancer usually results in the ability to produce AFP, but AFP level in some HAS is not high, and elevation of AFP can also occur in common gastric cancer with intestinal metaplasia. The elevation of serum AFP is not a justified criterion for the diagnosis of HAS. However, studies have shown that elevated AFP level is related to prognosis of patient [13]. In our study, 41 patients (83.7%) had elevated AFP, and 8 patients (16.3%) had normal AFP. The maximum serum AFP was 7335 ng/ml. We defined AFP positive as patients with more than 500 ng/ml serum AFP. But there was no significant statistical difference in AFP positive rate between the high-risk group and the low-risk group (*P* = 0.074). The relation of elevated AFP level between prognosis for HAS patients requires further verification with greater sample sizes.

In this study, multivariate analysis showed that serum CEA level was an independent prognostic factor for patients with HAS undergoing radical surgical resection (*P* = 0.018), and serum CEA > 5.0 ng/ml before treatment suggested poor prognosis. It has been reported that serum CEA level is related to tumor size, local invasion, lymph node and liver metastasis, and serum CEA level increases with the increase of clinical stage. This may be related to the enhancement of tumor invasion by the adhesion, immunosuppression and protease inhibition properties of CEA. Meanwhile, CEA, as endogenous immunosuppressant and protease inhibitor, can inhibit host-specific and non-specific immune responses throughout the whole process of tumor metastasis.

In some studies, imaging and pathological features were combined, and the results showed that greater vascular invasiveness was one of the characteristics of HAS [14]. HAS have a higher incidence of vascular invasion than common gastric adenocarcinoma, which is associated with a poor prognosis [11, 15, 16]. In our study, the results showed that vascular tumor thrombectomy was an independent factor affecting the prognosis of patients (*P* = 0.004). The occurrence of vascular tumor thrombi is closely related to the invasive characteristics of the tumor, and its existence is not isolated, usually associated with neurovascular bundle and lymphatic invasion [17]. Studies have found that the presence of vascular carcinoma thrombus is closely related to lymph node metastasis, and the tumor cells in lymph vessels around the lesion correspond to the invasion of the lymph system by tumors [18]. Vascular tumor thrombus extends from tumor to mesenteric vessels and is a necessary step to start the metastatic cascade [19], which allows tumor cells to embolize through the portal vein circulation, leading to distant metastasis of HAS.

Multivariate analysis results showed that among the CT imaging factors of HAS before treatment, peritumor fat space infiltration was an independent CT imaging risk factor affecting the prognosis of HAS patients as well. Fuzzy primary serosal surface and surrounding fat infiltration suggest higher T stage of tumors and stronger invasiveness to surrounding tissues, thus leading to worse clinical prognosis. Differentiating between T3 and T4a using CT is very difficult because the gastric serosa is not delineated on CT images. Conventional CT scans evaluate serosa penetration by observing the density of the peritumoral fatty space. However, relying solely on this criterion may lead to overstaging, attributable to various factors that change the peritumoral fatty space, such as tumor invasion, perigastric inflammation, and reactive fibrous connective tissue hyperplasia. [20–23]. In our study, peritumoral fat space infiltration was defined as a substantial density increase of the peritumoral fat space or the presence of nodular or irregular linear opacities in the peritumoral fatty space. However, slight blurring and a “smudged” appearance were excluded to obviate the influence from inflammation and fibrosis. Previous pathological studies on gastric cancer have found that isolated cancer cells and cancer nodules separated from primary foci and lymph nodes exist in gastric mesangial adipose tissue of postoperative gastric cancer specimens, and this fatty infiltration has been considered to be related to the invasiveness of the disease, which may be caused by the direct diffusion of primary tumor cells, or may be related to the lymph-venous communication formed by metastatic lymph nodes, and is one of the independent risk factors for poor prognosis of gastric cancer patients [24]. Additionally, in this study, the proportion of cases of esophagogastric junction carcinoma and gastric antrum was relatively high, and the proportion of cases of esophagogastric junction carcinoma was 34.7% (17 patients), higher than the proportion reported in the literature (28.3%, 15/53) [24]. Univariate analysis results of this study indicated that the proportion of patients with primary lesions located at the esophagogastric junction and cardia, as well as located at the gastric body, had a significant statistical difference between the high-risk group and the low-risk group (*P* = 0.046). Since the esophagogastric junction is not covered by the serous membrane, the presence and extend of tumor component infiltration in the fatty connective tissue around the lesion cannot be precisely reflected in the T stage. Thus, esophagogastric junction tumor with peritumoral fat infiltration are more likely to be evaluated as T3 stage without any difference. Therefore, the prognostic risk of these T3 esophageal and gastric junction carcinoma cases with peripheral fat infiltration may be underestimated potentially leading to varying impacts on the prognostic assessment of different risk subgroups [20, 23].

There were some limitations in this study. Firstly, the sample size was small due to the rarity of HAS patients undergoing radical surgical resection. There were relatively too many variables included in the analysis given the small sample size, which could potentially impact the results. Second, it was retrospective and enrolled patients in a single institutional cohort, some inevitable issues, such as information biases, might exist owing to its retrospective design. Third, the treatment regimens of enrolled patients in the study were not uniform. In this study, elevated serum CEA level, peritumoral fatty space invasion and positive pathological vascular tumor thrombus were found as independent factors for disease recurrence. Perspective and multicenter research with a larger sample size is warranted for validating the findings of this study, meanwhile, radiomics will be helpful for future research. Additionally, subgroup analysis could help detect if there are different factors influencing recurrence for the specific subgroups of HAS patients.

In conclusion, preoperative CT appearance of peritumoral fatty space invasion, elevated serum CEA level, and pathological vascular tumor thrombus indicated poor prognosis of HAS patients.

### Supplementary Information

**Additional file 1:** **Table S1.** Adjuvant and neoadjuvant regimens.

## Data Availability

The datasets used or analyzed during the current study available from the corresponding author on reasonable request.

## Abbreviations

HAS: Hepatoid adenocarcinoma of stomach
AFP: α-Fetoprotein
CEA: Carcinoembryonic antigen
