# Supplementary material for: Analysis of enhanced CT imaging signs and clinicopathological prognostic factors in hepatoid adenocarcinoma of stomach patients with radical surgery: a retrospective study
Source: BMC Med Imaging. 2023 Oct 26;23:167. doi: 10.1186/s12880-023-01125-z (PMC10604919; doi:10.1186/s12880-023-01125-z)
Supplement: Supplementary file 1 — Additional file 1: Table S1. Adjuvant and neoadjuvant regimens. [file 12880_2023_1125_MOESM1_ESM.docx]

| **Table S1.** Adjuvant and neoadjuvant regimens | | | | |
| --- | --- | --- | --- | --- |
| **Neoadjuvant therapy** | **Neoadjuvant regimen** | **Postoperative adjuvant therapy** | **Regimen of postoperative adjuvant therapy** | **Recurrence** |
| + | SOX | + | SOX | - |
| + | DCF | + | DCF | - |
| + | XELOX | + | SOX | - |
| + | SOX | + | SOX | - |
| + | XELOX | + | XP | - |
| + | XELOX | + | XELOX | - |
| + | SOX | + | SOX | - |
| + | XELOX | + | XELOX | - |
| + | SOX | + | Oxaliplatin+Tegafur | + |
| + | POS | + | Paclitaxel | + |
| + | Paclitaxel+Capecitabine | + | Paclitaxel | + |
| + | Capecitabine | + | Capecitabine | + |
| + | SOX | - |  | + |
| + | POS | - |  | + |
| + | SOX | + | Tegafur | + |
| + | POS | + | SOX | + |
| + | Oxaliplatin+Raltitrexed | + | Paclitaxel+Oxaliplatin | + |
| + | SOX | + | SOX | + |
| + | XELOX | + | Paclitaxel+Tegafur | + |
| - |  | + | XELOX | - |
| - |  | - |  | - |
| - |  | + | SOX | - |
| - |  | + | SOX | - |
| - |  | + | SOX | - |
| - |  | + | SOX | - |
| - |  | + | SOX | - |
| - |  | - |  | - |
| - |  | + | Oxaliplatin+Tegafur | - |
| - |  | + | Paclitaxel+Capecitabine | - |
| - |  | - |  | - |
| - |  | + | XELOX | - |
| - |  | + | SOX | - |
| - |  | + | SOX | - |
| - |  | - |  | - |
| - |  | + | SOX | - |
| - |  | + | XELOX | + |
| - |  | + | SOX | + |
| - |  | + | Radiotherapy | + |
| - |  | + | SOX | + |
| - |  | + | SOX | + |
| - |  | + | SOX | + |
| - |  | + | POS | + |
| - |  | + | Radiotherapy+SOX | + |
| - |  | + | XELOX | + |
| - |  | - |  | + |
| - |  | + | Radiotherapy+XELOX | + |
| - |  | + | Radiotherapy | + |
| - |  | + | Capecitabine | + |
| - |  | + | XELOX | + |
| *** SOX,Oxaliplatin+Tegafur; XELOX, Oxaliplatin+Capecitabine; DCF, Docetaxel+Cyclophosphamide+Fluorouracil; POS, Oxaliplatin+Capecitabine; XP,Cisplatin+Capecitabine;** | | | | |
